# ^222^Rn and ^226^Ra concentrations in selected shallow circulation groundwaters from the Fore-Sudetic Monocline area

**DOI:** 10.1007/s10653-023-01496-w

**Published:** 2023-02-10

**Authors:** Piotr Maciejewski, Agata Kowalska

**Affiliations:** Faculty of Geoengineering, Mining and Geology, Wrocław University of Science and Technology, Na Grobli 15, 50-421 Wrocław, Poland

**Keywords:** Radon ^222^Rn, Radium ^226^Ra, Groundwaters, Fore-Sudetic Monocline

## Abstract

Natural radioactive isotopes occur in various components of the natural environment, including groundwater. The general population, not always aware of possible threats, can use its resources. The activity concentration of some of the radioactive isotopes should be monitored, especially in those intakes from which it is possible to obtain water for human consumption, e.g. in domestic wells. The conducted research was innovative due to the fact that in many countries, including Poland, there are no regulations on waters exploited from home wells using as a drinking water source. As the groundwaters from this area have not been examined for radon (^222^Rn) and radium (^226^Ra) occurrence yet, the goal of this research was to perform screening tests in this part of the Fore-Sudetic Monocline. For this purpose, the authors have measured the concentration of ^222^Rn and ^226^Ra activity in groundwater collected from this geological unit located in south-western Poland. ^222^Rn and ^226^Ra occurrence was researched, and specific electrolytic conductivity, redox potential, pH and temperature were measured in 52 groundwater samples. ^222^Rn activity concentration ranged from 0.18 to 19.78 Bq/dm^3^. Only in three cases, ^226^Ra activity concentration reached a value above the lower detection limit of the applied method, i.e. 0.05 Bq/dm^3^ (max. 0.77 Bq/dm^3^). The authors present completely new data on the occurrence of these radioactive isotopes in the waters of the first aquifer in the Fore-Sudetic Monocline, which is not without significance for the health of consumers of these waters.

## Introduction

The term *Fore-Sudetic Monocline* was introduced by Tokarski (1958). It refers to a geological unit located in the south-western part of Poland. It extends over the Polish national border into Germany in the west, borders on the Kraków–Częstochowa Monocline in the south-east, is bounded by the Middle Odra fault in the south-west and is bounded by the Kalisz–Poznań line in the north. As a geological unit, the Fore-Sudetic monocline is all covered by Tertiary and Quaternary deposits (Stupnicka, 2013). In terms of ordinary groundwater regionalization according to hydrogeological units, the Fore-Sudetic Monocline lies in the following regions: Greater Polish region, Lower Silesian region, Mogilno–Łódź–Nida region, Kraków–Częstochowa Jurassic Upland and the Silesian Triassic region (Paczyński & Sadurski, 2007).

Groundwaters from the Fore-Sudetic Monocline have not yet been the subject of geochemical analyses with regard to the presence of radon-222 (^222^Rn) and radium-226 (^226^Ra) isotopes. In Poland, such routine analyses are only conducted in waters intended for human consumption supplied to households by waterwork systems (Regulation, 2017) and those utilized as medicinal waters (Regulation, 2006). Lack of interest in the geochemistry of natural radioactive isotopes dissolved in waters in this region may be linked to its geological makeup which does not hint at increased activity concentrations of these isotopes. The main research into ^222^Rn and ^226^Ra occurrence in groundwaters has been centred on the areas with the presence of crystalline (igneous and metamorphic) rocks at small depths. These rocks are often rich in uranium, which, according to radioactive decay law, is the source of ^226^Ra, whose decay product is the longest lived (over 3.82 days) isotope of radon—^222^Rn. Although ^222^Rn and ^226^Ra belong to the same radioactive series, they do not often co-occur in groundwaters because of their hydrochemical characteristics. Except for radioactive decay of dissolved ^226^Ra^2+^ ions, the source of radon in groundwaters is chiefly direct emanation of gaseous ^222^Rn from rocks (Przylibski, 2005). There are numerous unpublished and published sources (Przylibski, 2005; Przylibski et al., 2007; unpublished PhD dissertation: Adamczyk-Lorenc, 2007, The hydrogeochemical background of radon in groundwaters in the Sudetes; Przylibski et al., 2008; Walencik et al., 2010; Przylibski & Górecka, 2014; Domin & Przylibski, 2014; unpublished PhD dissertation: Kowalska, 2017, Relation between increased ^226^Ra and ^228^Ra activity concentration occurrence and groundwater composition and chemical type; unpublished PhD dissertation: Domin, 2018, Potentially medicinal radon waters of the Fore-Sudetic block) presenting research results from areas whose geological makeup determines the occurrence of groundwaters with increased natural radioactivity related to the presence of radon in shallow waters. Due to lack of data on radon content in groundwaters in the Fore-Sudetic Monocline area, the presented analyses constitute a kind of geochemical prospection—the authors conducted hydrogeochemical screening of this area. The rocks building the youngest (Quaternary) deposits include those accumulated during the Pleistocene glaciations, so they can locally contain pieces of uranium-bearing rocks, i.e. granites or granitoids. This may, in turn, be reflected in local anomalies concerning radium and radon occurrence in groundwaters. The undertaken research centres on waters in the uppermost aquifer, which are normally not used in public wells and consequently are not subject to frequent quality control. However, they are often used for household purposes by residents who capture these waters in domestic wells, which makes checking the quality of such waters necessary. Additionally, it is in waters from the uppermost aquifer, i.e. shallow circulation waters, where ^222^Rn activity concentration is higher than in waters from deeper aquifers (Przylibski, 2011).

In Poland, like in the rest of Europe and many countries around the world (Przylibski, 2018), radon presence in water makes it unsuitable for direct consumption. Local laws, as well as the World Health Organization (WHO), specify the allowable concentrations of radon and other radioactive isotopes whose exceeding makes waters unsuitable for human consumption. On the other hand, according to the radiation hormesis theory, radon waters with an appropriately high ^222^Rn concentration may be used for medicinal purposes. Among the naturally occurring radioactive isotopes, radon is the only one that, on the one hand, is considered to have toxic properties as a carcinogen and, on the other hand, is sometimes considered to have a beneficial effect on human health. The radiation hormesis theory claims that small ionizing radiation doses may have beneficial (therapeutic) effect on the human organism (Calabrese & Baldwin, 2002, 2003; Erickson, 2007; Johansson, 2003). Based on this theory, radonotherapy treatments are offered in many health resorts, spas and natural treatment centres in Europe (e.g. in Germany, Austria, the Czech Republic, Poland, Hungary, Italy, Romania, Greece and Russia), Asia (in Japan and China), North America (the USA) and South America (Chile). In Poland, the level of radon (^222^Rn) activity concentration in groundwaters at which waters may be classified as having pharmacodynamic (therapeutic or radon) properties is 74 Bq/dm^3^ (Ustawa, 2011). It must be remembered, however, that although medicinal waters are abstracted in compliance with the local Geological and Mining Law (Ustawa, 2011) and they are treated as a mineral resource having an advantageous effect on human health, radon is still regarded as a factor causing respiratory tract tumours (IARC, 1988). This is the reason why its concentration in utility waters must be scrupulously monitored so the dose received by a user (consumer or patient) will not exceed the admissible annual effective dose. The WHO guidelines (WHO, 2017) and national regulations (Regulation, 2017) specify the maximum radon content in water intended for human consumption, which is 100 Bq/dm^3^. The presence of other radioactive isotopes and other harmful substances must be also continuously controlled if such waters are to be used in industry or balneotherapy (Regulation, 2006).

Despite the absence of symptoms pointing to the occurrence of increased activity concentrations of ^222^Rn (radon medicinal waters) and ^226^Ra in the indicated area, the authors decided to sample the area due to the likely presence of waters with radon and radium concentrations that might not necessarily be extreme but could make safe usage of these waters by the inhabitants of particular regions impossible.

When supplying water to people, increased ^222^Rn concentrations should be removed from waters before they are further distributed and consumed.

## Characteristics of the studied area

**Fig. 1:**
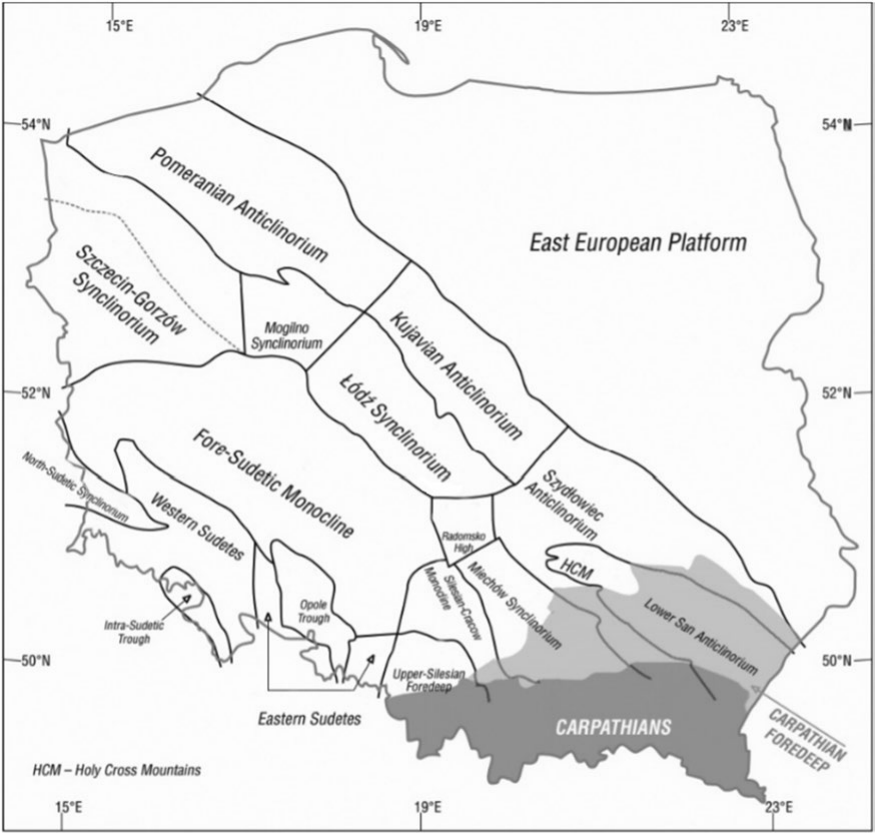
The main tectonic units of Poland on the sub-Cenozoic surface (Hycnar et al., 2021)

The Fore-Sudetic Monocline is situated in south-western Poland, and it is classified as a Mesozoic unit (Stupnicka, 2013). Its boundaries are contractual, and they run along intersection lines. It has been set apart from a wing of Szczecin–Łódź–Miechów basin (Fig. 1), and it has been pointed out (Dadlez & Marek, 1974; Dadlez, 1974a, 1974b) that the boundaries should be determined by taking account of deep-lying faults continued in the cover rocks. The monocline strata dip towards the north-east at a small angle of a few degrees at the most. The oldest rocks in this area are Permian rocks, which become thinner towards the south-east. Also, some Mesozoic strata wedge out partially. The rocks of the sub-Permian bedrock, as well as Permian and Triassic sediments, are not very thick. The Permian bedrock contains some Palaeozoic structures of the northern part of the Variscides of western Poland, including several fold units with considerable sizes. In the south, there is Rawicz synclinorium, which is built of unmetamorphosed Lower Permian and Namurian greywacke and schist deposits. To the north lies Leszno anticlinorium built of metamorphic schists (Stupnicka, 2013). In the northern part of the Fore-Sudetic Monocline, in Poznań synclinorium, there are Carboniferous rocks (Wierzchowska-Kicułowa, 1984).

Permian rocks are fluvial, limnic or aeolian Rotliegend deposits. They are overlain by volcanic rocks with thicknesses reaching even 500 m (Stupnicka, 2013) in places.

Starting in the Zechstein, subsidence occurred in the monocline area. Sedimentation was the fastest in Zielona Góra depression, while Leszno swell was a shoal formed of a Zechstein sediment facies, on which reef limestones were formed during emergencies (Peryt et al., 1978). As subsidence was smaller in the south-east, this is in this direction where Zechstein, Triassic and Jurassic sediments gradually recede (Stupnicka, 2013). Because of marine transgressions from the west, sediment insertions with marine fauna (e.g. in bunter sandstone and Keuper) are common in the Fore-Sudetic Monocline area. The end of the Jurassic period brought tectonic movements which led to the emergence of older Cretaceous deposits, subsequently covered with carbonate rocks in the Late Cretaceous. Upper Cretaceous deposits have only been preserved in in the form of the Opole Island. The area was later covered by Tertiary sediments.

From the point of view of the described research, however, these are the deposits overlying the Fore-Sudetic Monocline which are the most important, not those directly building it. Due to the fact that samples were collected from shallow-lying groundwaters, usually in the uppermost aquifer, it should be emphasized that they only had had contact with deposits reaching the depths of about a dozen meters below ground level at the most. These are mainly Quaternary sandstones, clays, gravels, river silts, sandstones, siltstones, silts, loesses, fluvisols, peats or aggregate muds. They might contain Quaternary rock material originating in the Middle Polish glaciation. The southern extent of the youngest of the Middle Polish glaciations, the Warta glaciation, is marked by moraines on the Dałkowskie and Trzebnickie Hills lying in the south-western part of the Fore-Sudetic Monocline. Their southern slopes are covered by diluvia, fluvioglacial sands and loesses. The rock material building them may be enriched with crystalline rocks accumulated by the Scandinavian ice sheet, with increased concentrations of numerous elements, including uranium and radium. Increased concentrations of various elements in younger deposits are due to the shorter period of them being leached by rainwater (Karpińska et al., 2002; Lis & Pasieczna, 1995; Tomaszewski, 1978). The result of these processes might be the appearance of local radon anomalies in this area. Moreover, large lithological variation, especially in tills, often clayed in one place and sand-rich in another, and the resultant differences in their permeability, may also contribute to local differences in radon concentration in soil air and in groundwaters circulating in this medium (Karpińska et al., 2002). These determinants also prompted the authors to undertake the research.

**Fig. 2:**
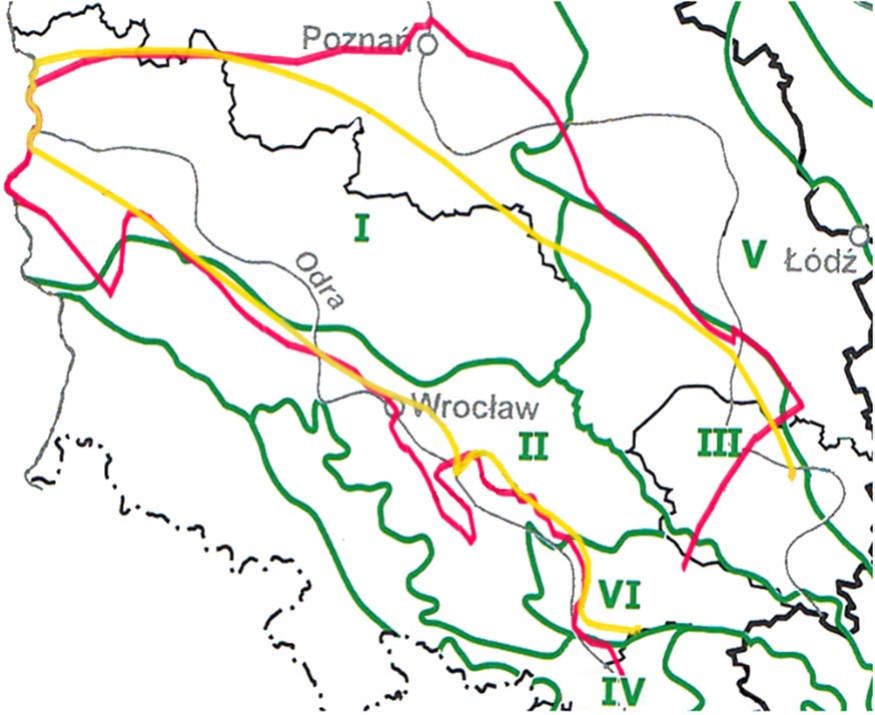
Division according to AHP hydrogeological units (Paczyński, 1995, altered). The boundary of the Fore-Sudetic Monocline according to Żelaźniewicz et al. (2011) (yellow line) and Narkiewicz and Dadlez (2008) (red line) on the base map of regional division of ordinary groundwaters (Paczyński & Sadurski, 2007). Regions: I—Greater Polish, II—Lower Silesian, III—Kraków–Częstochowa Jurassic, IV—submontane, V—Mogilno–Łódź–Nida, VI—Silesian Triassic

According to the division into hydrogeological units proposed by the Hydrogeological Atlas of Poland (AHP, Paczyński, 1995), the area of the Fore-Sudetic Monocline is divided between the following regions (Fig. 2.): Greater Polish, Lower Silesian, the Kraków–Częstochowa Jurassic region, the Silesian Triassic region and, additionally, the Mogilno–Łódź–Nida region (a small area in the south-east) and the submontane region (a small tip in the south).

## Methodology

**Fig. 3:**
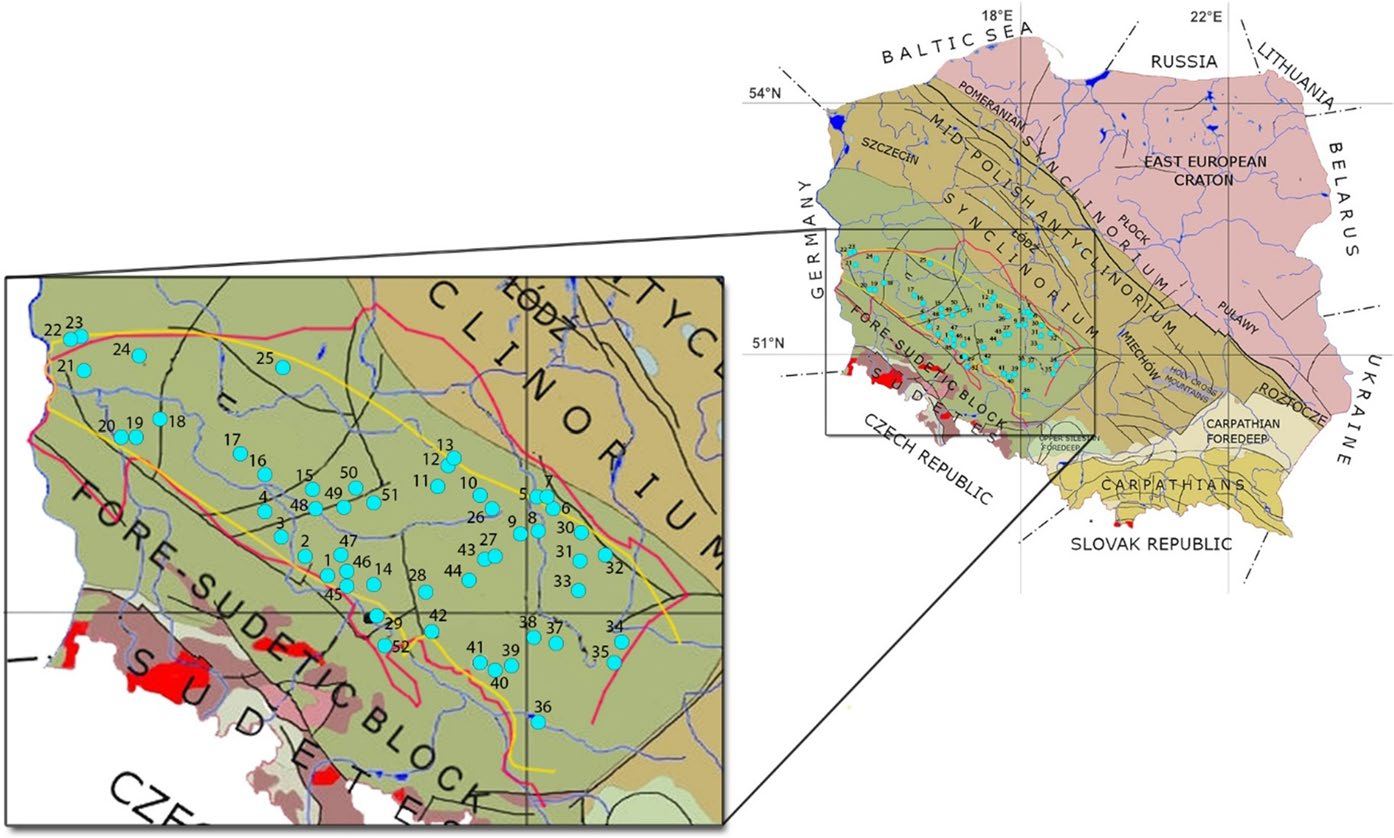
Water collection points in the Fore-Sudetic Monocline area (Znosko, 1998): red line—boundary according to Narkiewicz and Dadlez (2008), yellow line—boundary according to Żelaźniewicz et al. (2011); 1—Żerkówek, 2—Wodniki, 3—Tymowa, 4—Krzydłowice, 5—Godziesze Wielkie, 6—Aleksandria, 7—Saczyn, 8—Grabów nad Prosną, 9—Przedborów, 10—Lamki, 11—Krotoszyn, 12—Trzemoszno, 13—Grębów, 14—Wisznia Mała, 15—Kłoda Górowska, 16—Szlichtyngowa, 17—Krzepielów, 18—Zielona Góra, 19—Piaski, 20—Bogaczów, 21—Radomicko, 22—Radzików, 23—Mierczany, 24—Węgrzynice, 25—Ruchocice, 26—Wysocko Małe, 27—Bierzów, 28—Boguszyce, 29—Wrocław, 30—Brąszewice, 31—Kopaniny, 32—Złoczew, 33—Biała-Kopiec, 34—Kleśniska, 35—Starokrzepice, 36—Pustków, 37—Maciejów, 38—Rożnów, 39—Miejsce, 40—Zieleniec, 41—Święciny, 42—Sątok, 43—Myślniew, 44—Nowy Dwór, 45—Niziny, 46—Osolin, 47—Strupina, 48—Lechitów, 49—Załęcze, 50—Zakrzewo, 51—Chojno, 52—Osiek

In order to perform the planned screening for ^222^Rn and ^226^Ra occurrence in groundwaters of the Fore-Sudetic Monocline, 52 groundwaters samples were taken from the uppermost aquifer. The sampled intakes were wells, to a large extent domestic ones (supplying individual households). The precise locations of the sampled sites were determined (Fig. 3) with a sensitive dual-band receiver tracking GPS signal and GLONASS—GPS Garmin Monterra™. Additionally, basic physico-chemical parameters of water: the oxidation–reduction potential (Eh), pH, specific electrolytic conductivity (SEC) and the temperature (T), were measured using a Multi 3430 model of the multi-parameter WTW MultiLine meter. The meter is equipped with three measurement channels. A pH measurement using this meter is possible in the range from 0.000 to 14.000 ± 0.004 at a temperature from 0 to 100 °C. The reference electrolyte is KCl at a concentration of 3 mol/dm^3^. The accuracy of temperature measurements is ± 0.2 °C. The SEC was measured with a conductometer probe in the range from 10 to 2000 μS/cm, with the accuracy ± 0.5%. The Eh was measured using a glass shaft electrode with the measurement range from − 1200.0 to 1200 mV and the accuracy of up to 0.2 mV (Catalogue, 2019).

Ten cm^3^ water samples were collected with calibrated BD Discardit™ II syringes and KDM® KD-FINE® needles to glass vials made of potassium-depleted glass (in order to limit the concentration of the radioactive isotope ^40^K). Previously, in the laboratory, each vial was filled with 10 cm^3^ of Insta-FLUOR™ Plus scintillator. The collected water sample was injected under the scintillator layer and, after sealing the vial, shaken vigorously, which enabled the passage of gaseous radon contained in water to the scintillator (radon solubility is higher in the organic phase than in the aqueous phase). Additionally, the precise time of sampling was recorded. This time was used for calculating ^222^Rn and ^226^Ra activity concentrations. The sample prepared in this way was transported to the Earth Sciences and Mineral Engineering Laboratory at Wrocław University of Science and Technology. In the laboratory, ^222^Rn and ^226^Ra activity concentrations were measured with an ultra-low background liquid-scintillation spectrometer *α*/*β* Quantulus 1220 supported by WinQ software.

The spectrometer was equipped with a scintillation detector, whose operation is based on the phenomena of scintillation and external photoelectric effect. Using the differences in the duration of particular pulses and thanks to the appropriately selected parameters, the device distinguishes alpha particles from beta particles. Pulse shape analysis (PSA) enables registration of alpha and beta spectra and identifying the particles which have caused the pulse. Additionally, the spectrometer enables the reduction of beta radiation background. Background is also reduced by means of an anti-coincidence shield (Instrument manual, 2002; unpublished PhD dissertation: Kowalska, 2017, Relation between increased ^226^Ra and ^228^Ra activity concentration occurrence and groundwater composition and chemical type). Both the sample and the detector are shielded from the impact of radiation sources which do not originate in the substance in the measuring vial. It is very important, especially as the studied water samples from the Fore-Sudetic Monocline were not expected to contain high values of ^222^Rn and ^226^Ra activity concentrations, which made it necessary to employ a method that would enable obtaining the lowest possible detection limit.

During the measurement, a constant temperature of 18.2 °C was maintained inside the spectrometer. The measurement started about five hours after the vials was placed inside the measurement chamber of the spectrometer. This was to achieve a constant measurement temperature, as well as to put out the flares resulting from exposure to white light. The main goal, however, was to obtain radioactive equilibrium between ^222^Rn and its short-lived daughters. The device was programmed so that pulses from a single vial would be counted nine times and each time would last 60 min. In this way, 27 results were obtained from one sampling site. After conducting statistical analysis and rejecting outliers, the weighted mean of the number of registered pulses was calculated.

The above measurements of radon activity concentration do not damage the samples. Therefore, they can be further used to measure ^226^Ra activity concentration. After the time necessary to determine the radioactive equilibrium between ^226^Ra and ^222^Rn, and after the time ensuring the decay of radon originally dissolved in water to a concentration lower than the detection limit, a renewed measurement of radon activity concentration is possible. However, after the necessary time has passed, radon dissolved in a water sample originates entirely from the decay of ^226^Ra nuclei present in the sample, and the ^222^Rn activity concentration obtained by calculation is equivalent to ^226^Ra activity concentration.

For both ^222^Rn and ^226^Ra activity concentration measurements, measurement uncertainty was defined as complex uncertainty for uncorrelated measured parameters. It was the root of the sum of the products of squared partial derivatives and squared standard deviations of the mean values for: the number of alpha particle counts, water sample volumes and the correction coefficient.

The values of radon activity concentration collected for the studied geological unit were used for determining the range of hydrogeochemical background of ^222^Rn. This required several operations aimed at verifying the available data and analysing the type of statistical distribution of these values. The methodology of this procedure was described elsewhere (Przylibski et al., 2020). At the assumed significance level (0.05), the hypothesis of normal and log-normal distribution was rejected. At the significance level of 0.04, the distribution is consistent with normal distribution. As the normal distribution hypothesis was rejected at the originally assumed significance level (0.05), the hydrogeochemical background was calculated in two ways. The most reliable method is the computational method *Z* ± 1.28*σ*, where *Z* is the mean value and *σ* the standard deviation (Adamczyk-Lorenc, 2007). The other way of calculating the background, usually used when data distribution is not a normal (or log-normal) distribution, is based on the median M and its standard deviation *σ*M (M ± *σ*M).

## Results and discussion

A total of 52 wells capturing groundwaters from the uppermost aquifer in the area of the Fore-Sudetic Monocline were selected for the study. An overview of the values of basic descriptive statistics for all the measured parameters of the studied groundwaters is given in Table 1.

**Table 1:** An overview of basic statistical parameters for all the performed determinations

	Number of measurements	Minimum	Maximum	Median	Arithmetic mean	Standard deviation
^222^Rn [Bq/dm^3^]	52	0.18	19.78	1.93	4.59	5.14
^226^Ra [Bq/dm^3^]	52	< 0.05	0.77	–	–	–
SEC [μS/cm]	52	290	2370	687	765	428
pH [−]	52	6.482	8.588	8.044	7.999	0.422
Eh [mV]	52	− 169.9	306.8	83.7	97.8	81.5
*T* [ °C]	52	17.4	7.1	11.1	11.0	2.3

The results of field measurements of basic physico-chemical parameters conducted at groundwater intakes are given in Table 2. They demonstrated the prevalence of alkaline waters prevailed among the studied groundwaters. The lowest pH value, 6.482 ± 0.005, was measured in Osiek. The maximum value was 8.588, measured in Niziny. The arithmetic mean of the obtained results was 7.999, with a standard deviation of 0.422. The Eh value oscillated in the range from − 169.9 ± 0.3 mV (Piaski) to 306.8 mV ± 0.3 mV (Wrocław). The mean Eh was 97.8 mV, with a standard deviation of 81.5 mV. Most studied waters demonstrated positive Eh values (Table 2). This might indicate that these are shallow circulation waters recharged with infiltration waters rich in atmospheric oxygen. The occurrence of negative values of redox potential may prove that neither atmospheric air nor oxygen-rich infiltration waters had access to reach these waters. Such waters might be deeper circulation waters, although this has not been proven by the measured SEC value (not necessarily high TDS values in these waters). On the other hand, not all deep circulation waters are characterized by increased TDS content. What is more, no increased concentrations of radium, mostly (although not always) characteristic of deep circulation waters, were recorded in these waters. The presence of radon in groundwater environment is determined by its geochemistry and the composition of waters in which it is dissolved (unpublished PhD dissertation: Kowalska, 2017, Relation between increased ^226^Ra and ^228^Ra activity concentration occurrence and groundwater composition and chemical type).

**Table 2:** Characteristics of studied groundwaters from the Fore-Sudetic Monocline area in terms of basic physico-chemical parameters: specific electrolytic conductivity (SEC), pH, oxidation–reduction potential (Eh) and temperature (T)

Collection date	No.	Location	SEC	pH	Eh	*T*
			[μS/cm]	[−] ± 0.005	[mV] ± 0.3 mV	[°C] ± 0.2 °C
14.02.2019	1	Żerkówek	435 ± 3	8.148	175.2	7.6
	2	Wodniki	290 ± 2	8.400	111.5	10.4
	3	Tymowa	667 ± 4	8.390	75.8	7.8
	4	Krzydłowice	440 ± 3	8.329	60.3	8.8
17.02.2019	5	Godziesze Wielkie	854 ± 5	8.217	153.9	11.1
	6	Aleksandria	504 ± 4	8.529	50.6	12.7
	7	Saczyn	1424 ± 8	7.910	174.0	8.9
	8	Grabów n. Prosną	584 ± 4	8.240	30.4	11.5
	9	Przedborów	363 ± 3	8.334	36.0	11.4
	10	Lamki	894 ± 5	8.428	157.9	11.2
	11	Krotoszyn	857 ± 5	7.510	43.9	11.8
	12	Trzemeszno	677 ± 4	8.286	158.4	9.3
	13	Grębów	685 ± 4	8.186	37.2	9.4
18.02.2019	14	Wisznia Mała	770 ± 5	8.036	147.4	10.3
	15	Kłoda Górowska	884 ± 5	7.915	248.7	13.1
	16	Szlichtyngowa	2110 ± 12	7.764	100.3	13.3
	17	Krzepielów	722 ± 5	8.352	163.2	11.1
19.02.2019	18	Zielona Góra	1160 ± 7	7.390	81.9	11.5
	19	Piaski	291 ± 2	8.287	− 169.9	11.4
	20	Bogaczów	321 ± 3	7.940	145.2	11.8
	21	Radomicko	367 ± 3	8.565	− 60.4	10.9
	22	Radzików	731 ± 5	8.187	195.3	9.7
	23	Mierczany	405 ± 3	8.399	153.6	8.4
	24	Węgrzynice	305 ± 3	8.086	97.6	11.1
	25	Ruchocice	785 ± 5	8.368	83.7	8.3
31.03.2019	26	Wysocko Małe	1312 ± 8	7.277	− 58.9	11.0
	27	Bierzów	378 ± 3	7.464	214.5	10.2
	28	Boguszyce	474 ± 3	8.040	229.2	10.9
	29	Wrocław	628 ± 4	7.847	306.8	13.0
13.04.2019	30	Brąszewice	957 ± 6	8.157	72.8	10.7
	31	Kopaniny	1079 ± 6	7.733	50.7	7.4
	32	Złoczew	901 ± 6	8.198	62.3	7.9
	33	Biała-Kopiec	2370 ± 13	7.989	79.7	7.8
	34	Kleśniska	598 ± 4	8.299	61.7	7.1
	35	Starokrzepice	1351 ± 8	7.927	81.7	7.9
14.04.2019	36	Pustków	380 ± 3	8.207	83.6	14.2
	37	Maciejów	841 ± 5	6.733	42.3	8.1
	38	Rożnów	912 ± 6	7.758	59.0	11.9
	39	Miejsce	517 ± 4	8.048	47.1	11.6
	40	Zieleniec	1289 ± 7	8.246	58.3	11.0
	41	Święciny	756 ± 5	7.940	62.9	9.9
	42	Sątok	524 ± 4	8.399	60.1	11.1
27.10.2019	43	Myślniew	293 ± 2	8.039	133.8	17.3
	44	Nowy Dwór	303 ± 3	7.195	38.7	17.4
28.10.2019	45	Niziny	688 ± 4	8.588	124.6	13.0
	46	Osolin	320 ± 3	7.913	109.2	13.1
	47	Strupina	915 ± 6	7.935	183.5	13.7
	48	Lechitów	676 ± 4	7.981	181.3	13.4
	49	Załęcze	547 ± 4	7.699	142.4	12.9
	50	Zakrzewo	1429 ± 8	7.772	170.6	14.7
	51	Chojno	920 ± 6	7.885	82.3	13.3
16.05.2020	52	Osiek	890 ± 5	6.482	238.0	10.9

The average temperature of groundwaters at the place of their abstraction was 11.0 °C ± 0.2 °C, and its standard deviation was 2.3 °C. The minimum temperature of water was 7.1 °C ± 0.2 °C (Kleśniska), and the maximum was 17.4 °C ± 0.2 °C (Nowy Dwór). The lowest SEC value, 290 μS/cm ± 2 μS/cm, was recorded in Wodniki, and the highest, registered in Biała-Kopiec, was 2370 μS/cm ± 13 μS/cm, which, converted to TDS (2), are 1.78 mg/dm^3^ dissolved solids (poorly mineralized water) (Pazdro, 1983). The arithmetic mean was 764 μS/cm, and the standard deviation was 428 μS/cm.

**Fig. 4:**
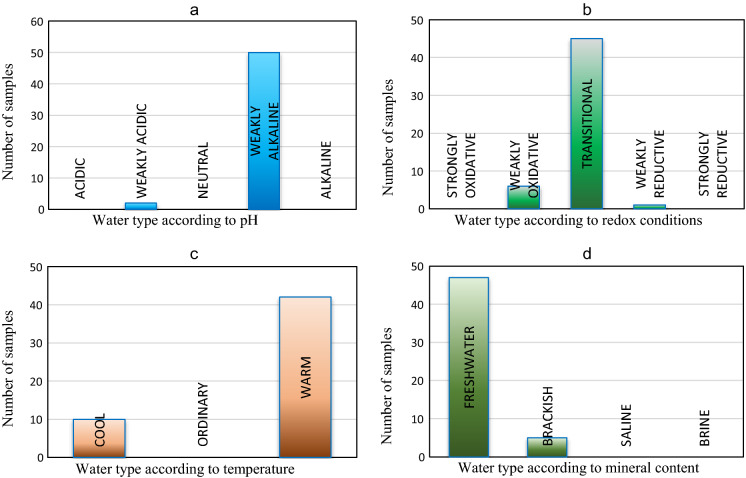
Histograms depicting the number of samples representing particular types of groundwaters according to: pH (**a**), oxidation–reduction conditions (**b**), temperature (**c**) and mineral content (**d**)

The above results can be interpreted in accordance with various classifications used in hydrogeology. According to pH-based classification (Witczak & Adamczyk, 1994), waters from the studied area are mostly weakly alkaline (50 intakes), and 2 are weakly acidic. No acidic, pH-neutral or alkaline waters were recorded in any of the sampled intakes (Fig. 4a). The puzzlingly high pH values indicating weak alkalinity in the majority of the studied waters may be due to the fact that the wells from which water samples had been collected were situated in the vicinity of farmland. Groundwater alkalinity may be related to relatively large concentrations of bicarbonates, carbonates, silicates or phosphates of alkaline earth minerals (Macioszczyk & Dobrzyński, 2002), but it may also be due to the presence of nitrates resulting from the use of fertilizers in the nearby fields (Kaniuczak & Augustyn, 2011). The positive Eh values suggest that the studied waters are largely shallow circulation waters with low TDS content which could be mixed with runoff waters leaching fertilizers from fields.

In order to classify these waters according to their redox conditions, a redox scale (Merkel & Sperling, 1996) was used and the results were converted according to formula (1):

1 $${\text{rH}} = \frac{{\left( {{\text{Eh}} + 0.06{\text{pH}}} \right)}}{0.03}$$

where rH—redox scale, Eh—measured value of oxidation–reduction potential, mV, pH—measured pH value.

The majority of results (45) point to transitional conditions, 6—weakly reductive conditions and only 1—weakly oxidative ones. Strongly oxidative or strongly reductive conditions were not found in any of the intakes (Fig. 4b).

According to a classification (Dynowska & Tlałka, 1982) comparing water temperature with mean annual air temperature, the water captured at 42 sites was warm and at 10 sites—cool (Fig. 4c). However, it must be remembered that the wells from which water was withdrawn might not have been exploited on a regular basis, so temperature measurements might have been subject to considerable error, and the conclusions—far-fetched. The measured temperatures were undoubtedly affected by the atmospheric air.

The last of the measured parameters was SEC. In this case, in order to classify the waters, the SEC was converted to total dissolved solids (TDS). Formula (2) was applied. Although literature sources (Kleczkowski, 1984; Macioszczyk & Dobrzyński, 2002) suggest using coefficient *f* values ranging from 0.52 to even 1, the value *f* = 0.75 was adopted for the calculations. This value is used in internal laboratory procedures for similar investigations, and it is based on long-term analyses of this kind.

2 $${\text{TDS}} = {\text{PEW}}_{25} \cdot f$$

where

TDS—total dissolved solids content in water,

SEC_25_—specific electrolytic conductivity compensated to 25 °C, S/cm^3^,

*f*—numerical coefficient, cm mg/S dm^3^.

According to the classification by Witczak and Adamczyk (1994), 47 groundwaters were freshwaters and 5—brackish waters. No saline waters or brines were identified (cf. histogram in Fig. 4d). According to classification by Pazdro (1983), the studied waters can be categorized as freshwaters with TDS content from 0.1 to 0.5 g/dm^3^ (22 samples), acratopegae with TDS content higher than that in freshwaters, but not higher than 1 g/dm^3^ (25 samples), and poorly mineralized waters (5 samples, TDS content 1.0–3.0 g/dm^3^).

Owing to lack of data concerning the occurrence of waters containing radon in the area of the Fore-Sudetic Monocline, the key part of the authors’ work were measurements of ^222^Rn activity concentration in samples taken from selected intakes. The obtained radon (^222^Rn) activity concentration results from the area of the Fore-Sudetic Monocline are collated in Table 3.

**Table 3:** Characteristics of the studied groundwaters from the area of the Fore-Sudetic Monocline in terms of radon (^222^Rn) and radium (^226^Ra) content

Collection date	No	Location	^222^Rn activity concentration	^226^Ra activity concentration
			[Bq/dm^3^]	[Bq/dm^3^]
14.02.2019	1	Żerkówek	0.32 ± 0.06	< 0.05
	2	Wodniki	1.84 ± 0.14	0.08 ± 0.02
	3	Tymowa	0.83 ± 0.09	< 0.05
	4	Krzydłowice	12.40 ± 0.45	< 0.05
17.02.2019	5	Godziesze Wielkie	7.76 ± 0.43	< 0.05
	6	Aleksandria	4.96 ± 0.34	< 0.05
	7	Saczyn	11.24 ± 0.55	< 0.05
	8	Grabów nad Prosną	0.68 ± 0.13	< 0.05
	9	Przedborów	8.09 ± 0.45	< 0.05
	10	Lamki	8.54 ± 0.47	< 0.05
	11	Krotoszyn	12.64 ± 0.61	< 0.05
	12	Trzemeszno	11.71 ± 0.58	< 0.05
	13	Grębów	4.51 ± 0.33	< 0.05
18.02.2019	14	Wisznia Mała	15.52 ± 0.75	< 0.05
	15	Kłoda Górowska	0.56 ± 0.16	< 0.05
	16	Szlichtyngowa	0.18 ± 0.14	< 0.05
	17	Krzepielów	2.09 ± 0.26	< 0.05
19.02.2019	18	Zielona Góra	7.61 ± 0.48	< 0.05
	19	Piaski	0.78 ± 0.17	< 0.05
	20	Bogaczów	15.76 ± 0.75	< 0.05
	21	Radomicko	2.58 ± 0.28	< 0.05
	22	Radzików	3.77 ± 0.54	< 0.05
	23	Mierczany	2.02 ± 0.36	< 0.05
	24	Węgrzynice	1.52 ± 0.31	< 0.05
	25	Ruchocice	4.48 ± 0.62	0.75 ± 0.11
31.03.2019	26	Wysocko Małe	1.09 ± 0.16	0.77 ± 0.11
	27	Bierzów	0.98 ± 0.15	< 0.05
	28	Boguszyce	0.69 ± 0.12	< 0.05
	29	Wrocław	1.75 ± 0.23	< 0.05
13.04.2019	30	Brąszewice	0.30 ± 0.11	< 0.05
	31	Kopaniny	4.55 ± 0.58	< 0.05
	32	Złoczew	15.34 ± 1.35	< 0.05
	33	Biała-Kopiec	0.33 ± 0.13	< 0.05
	34	Kleśniska	0.97 ± 0.23	< 0.05
	35	Starokrzepice	3.03 ± 0.47	< 0.05
14.04.2019	36	Pustków	0.42 ± 0.14	< 0.05
	37	Maciejów	14.86 ± 1.31	< 0.05
	38	Rożnów	1.15 ± 0.24	< 0.05
	39	Miejsce	2.70 ± 0.42	< 0.05
	40	Zieleniec	0.69 ± 0.18	< 0.05
	41	Święciny	0.62 ± 0.17	< 0.05
	42	Sątok	0.67 ± 0.18	< 0.05
27.10.2019	43	Myślniew	0.98 ± 0.25	< 0.05
	44	Nowy Dwór	0.99 ± 0.26	< 0.05
28.10.2019	45	Niziny	1.67 ± 0.33	< 0.05
	46	Osolin	1.18 ± 0.27	< 0.05
	47	Strupina	19.78 ± 1.80	< 0.05
	48	Lechitów	0.73 ± 0.21	< 0.05
	49	Załęcze	0.76 ± 0.24	< 0.05
	50	Zakrzewo	8.27 ± 1.07	< 0.05
	51	Chojno	4.26 ± 0.70	< 0.05
16.05.2020	52	Osiek	7.36 ± 0.61	< 0.05

**Fig. 5:**
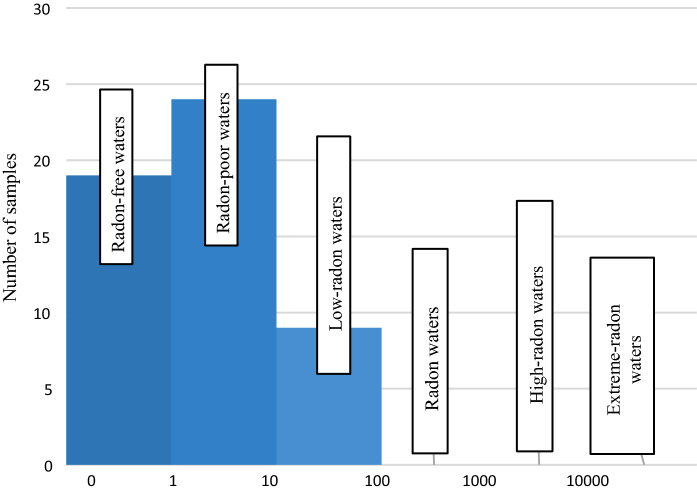
Division of the studied groundwaters from the area of the Fore-Sudetic Monocline with regard to the concentration of dissolved.^222^Rn, according to the classification by Przylibski (2005)

According to the classification by Przylibski (2005), groundwaters from 19 studied intakes can be categorized as radon-free waters (^222^Rn activity concentration below 1 Bq/dm^3^), those from another 24 intakes—as radon-poor waters (^222^Rn activity concentration from 1 to 9.9(9) Bq/dm^3^) and groundwaters from further nine intakes—as low-radon waters (^222^Rn activity concentration from 10 to 99.9(9) Bq/dm^3^) (Fig. 5).

The lowest ^222^Rn activity concentration was recorded in a groundwater sample from Szlichtyngowa and the highest—in one from Strupina, where it may have been caused by the closeness of the Silesian-Lubusian fault and the Sulmierzyce fault. The coefficient of radon emanation from reservoir rocks may be higher in this area, which might, in turn, cause increased ^222^Rn activity concentration in groundwaters in this area. It should be emphasized that this is only a hypothesis, and more detailed research would have to be conducted in order to confirm it. The mean ^222^Rn activity concentration for all the analysed samples was 4.59 Bq/dm^3^, with a standard deviation of 5.14 Bq/dm^3^ (Table 1).

**Fig. 6:**
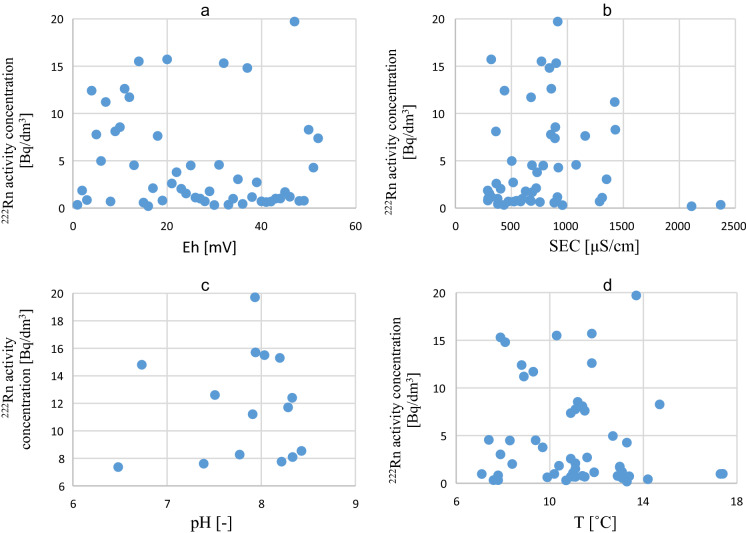
Correlations between ^222^Rn activity concentration and Eh (**a**), SEC (**b**), pH (**c**) and T (**d**)

On the basis of the available database, correlations between increased radon occurrence and particular physico-chemical parameters were studied. The study of these correlations did not confirm the existing clear relationship between ^222^Rn activity concentration in shallow circulation groundwaters and Eh, SEC, pH and T (Fig. 6a–d).

Radon concentration in the studied waters does not point to their therapeutic potential. In Poland, water classified as potentially medicinal radon water and, then, after satisfying a number of other requirements—medicinal radon water, is water with radon content of at least 74 Bq/dm^3^ (Ustawa, 2011). None of the analysed groundwaters meet this criterion. Additionally, none of the analysed samples was found to contain ^222^Rn activity concentration that would exceed the parametric value indicated in regulations concerning waters intended for human consumption (Regulation, 2017; WHO, 2017).

In view of the screening character of this research, a significant element of result analysis was the calculation of the hydrogeochemical background of radon for the uppermost aquifer in the area of the Fore-Sudetic Monocline. Graf’s test indicated the absence of outliers in the compiled database. Because of the rejection of the hypothesis of normal and log-normal distribution at the significance level 0.05, the background range was determined on the basis of the median M and its standard deviation *σ*M (M ± *σ*M). This was the range of 1 ÷ 7 Bq/dm^3^. The background value was calculated using the computational method *Z* ± 1.28*σ*, where *Z* is the mean value, which amounted to 0 ÷ 11 Bq/dm^3^. It must be remembered, however, that the hypothesis of normal and log-normal distribution was rejected at the assumed significance level (0.05).

Analyses of ^226^Ra activity concentration revealed that it was only in the waters from three localities: Wodniki, Ruchocice and Wysocko Małe where the value exceeded the method’s lower detection limit (LLD) equal to 0.05 Bq/dm^3^. The occurrence of waters with ^226^Ra activity concentration of 0.77 and 0.75 Bq/dm^3^ is an interesting problem, especially as these waters (judging by the measured redox potential) are not deeper circulation waters. Water samples from the wells in which the mentioned concentrations of ^226^Ra activity were determined were located at a considerable distance from each other. On the map, they are marked with numbers 26 and 25 (Fig. 1). In terms of radiology, such waters are not suitable for human consumption owing to the exceedance of the allowable concentration of this isotope, set by the Regulation (2017) at 0.5 Bq/dm^3^. Such local anomalies could be related to the rock material building the reservoir rocks of these waters. The source of radium-226 in the environment are isotopes of the uranium–radium series, chiefly ^238^U, ^234^U, or the more widespread though less soluble thorium (mainly ^230^Th). ^226^Ra enters the groundwater environment not only by the decay of its parent isotope (^230^Th), but also as a result of the weathering or dissolution of aquifer rocks containing thorium, uranium or radium (Jones & Atkins, 2004).

## Conclusions

Among the 52 analysed samples, radon-poor waters prevailed. In the light of the cited Polish regulations, all these waters satisfied the requirements specifying the ^222^Rn activity concentration allowed in waters intended for human consumption. The measured concentrations did not exceeded 100 Bq/dm^3^ in any of the sample, which means that these waters, in terms of radiochemistry (from the point of view of the presence of radon), do not constitute a health hazard for potential users. Also, none of the groundwater samples contained radon activity concentrations of more than 74 Bq/dm^3^, which would indicate the medicinal potential of studied waters. Based on the obtained results, ^222^Rn activity concentration does not depend on Eh, SEC, pH or T as well as water type defined on the basis of basic physico-chemical parameters. For the uppermost aquifer in the area of the Fore-Sudetic Monocline, it is relatively low.

The values of ^226^Ra activity exceeded the LLD only in three groundwater samples. The highest concentrations (0.77 Bq/dm^3^ and 0.75 Bq/dm^3^) may most likely be related to the rock material building the reservoir rocks. In the light of the current regulations, such waters cannot be intended for human consumption, as the parametric value is 0.5 Bq/dm^3^. These screening studies indicate that similar occurrences in other groundwater intakes in this geological unit should be expected in the future.

The obtained results constitute an original contribution to existing scientific knowledge in the area of natural radioactive isotopes in groundwater. The conclusions of the screening tests conducted lead to lobbying for the implementation of new legal regulations. Despite the low radon potential in the study area, the authors claim that it is worth monitoring all groundwater intakes from which people draw water, not only those that are part of the water supply networks.

## Data Availability

All data are presented in the article.
